# Effects of a Health Education Program on Fall Risk Prevention among the Urban Elderly: A Quasi-Experimental Study

**Published:** 2019-01

**Authors:** Piyathida KUHIRUNYARATN, Prasert PRASOMRAK, Bangonsri JINDAWONG

**Affiliations:** 1. Department of Community Medicine, Faculty of Medicine, Khon Kaen University, Khon Kaen 40002, Thailand; 2. Mahidol University Amnatchareon Campus, Amnatcharoen, Thailand

**Keywords:** Health education, Fall risk prevention, Elderly

## Abstract

**Background:**

Elderly falls increase dramatically with age and are a leading cause of injury, carrying a risk of loss of independence and death. We studied the effects of a health education program on fall-risk prevention among urban elderly persons in the municipality of Khon Kaen, Thailand.

**Methods:**

A quasi-experimental study was conducted in 2 communities. The calculated sample size was 216 individuals: 108 for intervention and 108 for control, all of whom were 60 or older, and registered at the Samlium Primary Care Unit (SPCU). The educational intervention was a fall risk intervention program by an elderly buddy. A structured questionnaire that incorporated questions from the Thai Fall Risk Assessment Tool (Thai-FRAT) was used to collect general and specific information. Data were analyzed using the independent sample t*-*test and χ^2^, with *P*<0.05 being statistically significant.

**Results:**

The response rate was 94.4%. More than half of the respondents were at risk of a fall. The prevalence of risk of a fall among the intervention group was slightly less than that for those within the control group [Intervention group=52.9% (95%CI: 42.85, 62.81, *P*<0.001); Control group=60.8% (95%CI: 50.59, 70.15, *P*=0.016)]. After 6 months of intervention, the balance impairment, medicine usage, and overall proportion with risk of fall were decreased. The difference between the intervention and control groups was statistically significant (*P*<0.05).

**Conclusion:**

The provision of a health education program designed for fall risk prevention among the elderly would be a useful public health initiative.

## Introduction

A fall is defined as an unintentional loss of balance, causing one to make unexpected and unprepared contact with the ground or floor (1). Falls are (a) the leading cause of injury-related visits to emergency departments in the United States and (b) the primary etiology of accidental deaths in persons over 65 (2). Falls have been known to contribute to an increased risk of premature death, disability, increased dependence, decreased social interactions, and premature nursing home admission (3).

The mortality rate for falls increases dramatically with age, regardless of race, sex, or ethnicity; with falls accounting for 70% of accidental deaths in persons 75 and older. Falls among elderly related to poor health, decline function, and morbidity. More than 90% of hip fractures occur as a result of falls, and most of these occur in persons over 70 (2). In addition, there exists a strong relationship between mobility, walking safety, and living independently in old age. People with walking problems suffer from a fear of falling, and tend to restrict their mobility and performance level within the community; even before a fall occurs (4). In Japan, falls are major public health problems and the second leading cause of death (within the subgroup of unintentional accidents) after road traffic accidents. Factors associated with falls in elderly were arthritis in the legs and taking at least 4 prescription medications daily. In addition, hemiplegia was found due to stroke associated with falls in men only (5).

In Thailand, the overall prevalence of falls among the elderly in an urban area was 19.8% during a 6-month period (6). Further examination revealed a prevalence of 24.1% in women but only 12.1% in men. When controlling for others significant factors the following were positively correlated to falls among the Thai urban elderly: regular medication (AOR 2.22, 95% CI 1.19, 4.12), depression (AOR 1.76, 95% CI 1.03, 2.99), sufficient exercise (AOR 0.34, 95% CI 0.19, 0.58), and wearing non-slip shoes (AOR 2.31, 95% CI 1.24, 4.29) (7).

Fall prevention requires the elimination of all possible fall risk factors. Using the concept of prevention and based on health belief model (8), prevention is defined as the planning for, and the measures taken, to forestall the onset of a disease or other problem before the occurrence of an undesirable health event (9). Health education is one of the most important community prevention programs for motivating people to adopt healthy behavior, develop positive attitudes, and to make decisions and incorporate the skills necessary to put their decision into practice. Thus, a health education program, which sets a combination of learning experiences -specifically designed to facilitate voluntary actions for the elderly-might be the most cost-effective, fruitful approach for reducing fall-risk (9). In this regard, interventions such as exercise programs and home safety interventions help to reduce the rate of falls (10).

The study setting, Khon Kaen in northeast of Thailand, has the second highest population in the Northeast region (1.7 million) of whom 13.8% are elderly (11). Few studies have focused on fall risk prevention programs, especially for persons residing in Khon Kaen Province. We aimed to study the effect of health education programs on fall-risk prevention among the urban elderly in Khon Kaen municipality.

## Materials and Methods

This was a quasi-experimental study carried out in the municipality of Khon Kaen. The Samlium Primary Care Unit (SPCU) has responsibility for 10 communities. This study purposively selected 2 communities for intervention and control. Data were collected (a) before the intervention and (b) 6 months after the intervention program. The inclusion criteria were persons: (a) at least 60 yr of age (b) registered for healthcare in the SPCU > 1 year. The exclusion criteria were elderly residents who were (a) dependent, (b) severely disabled, or (c) unable to participate or communicate with others in either chosen community. The elderly were recruited voluntarily each community.

### Sample size

The sample size was based on comparing the proportion of Fall-Risk Assessment for both groups in 2 group experiments. Pilot data suggested that the intervention group contained persons with a slightly lower risk of fall (41%) than that of the control group (60%). To detect a significant level (0.05, power 80%), the sample required 216 participants (108 in both the intervention and control groups). The recruitment of the elderly volunteers took place in both communities in Jan 2012.

### Ethics approval

Ethics approval for the study was obtained from Khon Kaen University. Upon invitation to the study, participants provided written consent. Their personal information was kept confidential.

### Intervention

The 6 months health education program on fall-risk prevention was specifically designed to reduce the possible barriers based on health believed model (8) for the learning process among the elderly including sensory loss, mental illness, and chronic diseases (12). Our health education program was elderly-centered; thus less lecturing and more interactive on-site training (13). Our program consisted of 3 parts: (a) fall risk prevention “buddy”, (b) home-based exercise, and (c) elderly group dialogue. The fall-risk prevention “buddy” was a training activity, in which one elderly participant chose another elderly participant to be his/her “buddy”. “Buddies” became their helpers and supporters, who monitored their behavior for fall prevention in 6 months of the study.

The content of the 1-day training for fall-risk prevention included factors related to falls including internal, external, and behavioral factors. Internal factors included (a) postural hypotension, (b) use of at least 4 prescription medications or sedatives, (c) impairment of arm or leg strength or range of motion, (d) impaired balance, transfer skills (inability to move safely from bed to chair, bathtub, or toilet), (e) gait disturbance, (f) history of stroke, (g) cognitive impairment, (h) visual impairment, (i) chronic pain, and (j) specific pathogenic ailments (14). External factors focused upon the home environment while behavioral factors included (i) consumption of alcohol, energy drinks, coffee or tea; (ii) smoking, (iii) insufficient exercise, (iv) walking up and down stairs daily, (v) rapid changes in posture, (vi) regular lifting of heavy objects, (vii) getting trapped in overly baggy clothes, and (viii) wearing shoes that slip easily (15).

In addition, a manual of fall-risk prevention, created by experts, was given to both the participant and his/her “buddy”. After completion of the training, monthly home visits were conducted with both participants and the research team. Discussions regarding the fall-risk prevention manuals, as well as additional posters and leaflets, were distributed to the community.

Home-based physical exercise aimed to strengthen and improve balance. There were 10 basic flexibility exercises performed at a low-intensity level; including warm-up, shoulder, arm, wrist, neck, leg, and back, followed by a cool-down. To prevent the risk of injury, physiotherapists first approved this exercise program then elderly participants implemented the program at a convenient place for 15–20 min a day.

The elderly group dialogue was design to offer the elderly participants an opportunity to share their fall-risk prevention activities and their experiences. Older participants would come to join the group dialogue—run by the elderly at the temple after finishing religious ceremonies every third week of the month. The process included sharing, telling of their experiences of falls and of fall-risk prevention for 15–20 min per person. The researchers observed the dialogue (set to run every 6 months).

There was no intervention in the control community. The activities in the control areas were conventional activities performed by health professionals responsible for those areas.

### Measurements

Measurements were taken at baseline then again after 6 months of intervention. The study tool was a questionnaire divided into 2 parts: (1) demographics and current health and (2) fall-risk assessment. The demographic and current health (including socio-demographic variables, chronic disease, and depressive symptoms) were assessed using questionnaire of the Geriatric Depression Scale (GDS, Thai version short form), with a total score of 15 (16). The cut-off was 6 or higher (16). Fall-risk assessment was measured using the Thai Fall-Risk Assessment Tool (Thai-FRAT) comprising 6 factors (including “History of falls”, “Impaired body balance”, “Female”, “Specific medication use”, “Impaired visual acuity”, and “Thai style house”). The possible score for the Thai-FRAT ranged between 0 and 11. The cutoff identified by the receiver operating curve analysis was 4 and over and the sensitivity and specificity were 0.92 and 0.83, respectively (17). The questionnaire was pre-tested and adjusted: the contents underwent some changes for better understanding of the elderly in Northeastern Thailand.

### Data collection and analysis

Trained interviewers collected data, using the questionnaire, checklist and medical records. The interviewers were trained to administer the questionnaire in a standardized fashion. One group of interviewers was used to interview pre- and post-intervention. The data abstraction and interview forms were checked for completeness, then double-entered and validated in the EPI INFO 6 before being transferred into SPSS (version 17, (Chicago, IL, USA) licensed to Khon Kaen University).

Descriptive statistics were used to describe sample characteristics, such as frequencies, percentages, Kolmogorov-Smirnov test, means, standard deviations, medians, IQRs, and 95% CIs, Chi-square, and McNemar’s test. A probability *P-*value<0.05 indicated a statistically significant relationship.

## Results

Overall, 204 elders agreed to participate in the study: the response rate was 94.4% in both the intervention and control groups.

### Socio-demographic characteristics

The elders (sample group) comprised more females than males, and the proportion of females was significantly higher in the control group than the intervention group. The mean age of the intervention respondents was only slightly higher than that of the control group (69.32 ±6.88 vs. 69.29±7.58, respectively). In terms of both education and marital status, there was again no statistically significant difference between the intervention and control groups.

In terms of health status and having a caregiver, there were only slight differences in the proportion of elderly respondents who had chronic diseases, perceived health status, and depressive symptoms between the intervention and control groups. We found that the proportion of the elderly who were cared for by family caregivers in the intervention group was slightly less than those in the control group (72.5% vs. 78.4%, respectively). In terms of chronic diseases and depressive symptoms, we found that the proportion in the intervention group was higher than in the control group (57.8% vs. 55.9% and 49.0% vs. 38.2%, respectively) (Table 1).

**Table 1:** Comparison of baseline demographics and health status characteristics between intervention and control groups

***Variables***	***Intervention n=102 n (%)***	***Control n=102 n (%)***	***P-value***
Sex			
Male	34 (33.3)	33 (32.4)	0.881
Female	68 (66.7)	69 (67.6)	
Age (yr)			
60–69	57 (56.4)	56 (54.9)	0.826
≥70	44 (43.6)	46 (45.1)	
Mean age in years (SD)	69.32 (6.88)	69.29 (7.58)	0.059
Education			
Uneducated	14 (13.7)	6 (5.9)	0.304
Primary school	72 (70.6)	77 (75.5)	
Secondary school	12 (11.8)	14 (13.7)	
Bachelor or higher	4 (3.9)	5 (4.9)	
Marital status			
Single	3 (2.9)	3 (2.9)	0.999
Married	65 (63.7)	64 (62.7)	
Widowed	32 (31.4)	33 (32.4)	
Divorced/separated	2 (2.0)	2 (2.0)	
Having a Caregiver			
Yes	74 (72.5)	80 (78.4)	0.329
No	28 (27.5)	22 (21.6)	
Chronic diseases			
Yes	59 (57.8)	57 (55.9)	0.777
No	43 (42.2)	45 (44.1)	
Depressive symptoms			
Yes	50 (49.0)	39 (38.2)	0.120
No	52 (51.0)	63 (61.8)	

* Chi-squared test

### Comparison of baseline fall risk between intervention and control groups

The baseline data showed no statistical difference between the intervention and control groups according to the Fall-Risk Assessment Tool (Thai-FRAT). When using a cut-off of 4, more than half of the elderly respondents were at risk of a fall.

The prevalence of the elderly at risk of a fall within the intervention group was slightly less than that of the control group (52.9% vs. 60.8%, respectively). The Thai FRAT mean score among the intervention group was only slightly less than that the control group (3.79±1.75 vs. 3.87±1.85) (Table 2).

**Table 2:** Comparison of baseline fall risk between intervention and control groups

	***Fall-Risk Assessment Tool (Thai-FRAT)***	***Intervention (n=102)***	***Control (n=102)***	***P-value***
	1. Females (%)	66.7	67.6	0.881
	2. Visual impairment (unable to read more than half of the letters in the 6/12 line of the Snellen chart) (%)	44.1	42.2	0.777
	3. Balance impairment (unable to take full tandem for 10 seconds) (%)	56.9	57.8	0.887
	4. Medical usage (taking 4 medications of any kind) (%)	48.0	51.0	0.674
	5. History of falls (fell ≥ 2 times in past 6 months) (%)	3.9	4.9	0.733
	6. Housing style (first floor 1.5 m or higher off ground: Traditional Thai style (%)	81.4	86.3	0.342
	Risk (≥ 4 score) (%)	52.9	60.8	0.258

* *statistical significance set at 0.05, Chi squared test*

### Effect of Health Education program for fall risk prevention and Thai-FRAT

Within the Thai-FRAT, the proportion of fall-risk was slightly decreased in the intervention group and of non-differential proportion in the control group. In both groups, the differences in the respective proportion of risk of balance impairment and medical use were statistically significant (Table 3). In terms of overall fall-risk, the difference in the decrease in the proportion of fall-risk was statistically significant between the intervention and control groups (Table 4).

**Table 3:** Within-group changes in fall risk after 6 months of intervention

***Fall-Risk Assessment Tool (Thai-FRAT)***	***Intervention (n=102)***			***Control (n=102)***						
	***Baseline***	***6 months after***	***P-value***	***Baseline***	***6 months after***	***P-value***
1. Females (%)	66.7	66.7	1.00	66.67	66.67	1.00
2. Visual impairment (unable to read more than half of the letters in the 6/12 line of the Snellen chart) (%)	44.1	44.1	1.00	42.2	42.2	1.00
3. Balanced impairment (unable to take full tandem for 10 seconds) (%)	56.9	32.1	<0.001	54.8	46.1	<0.001
4. Medical use (taking 4 medications of any kind) (%)	48.0	32.9	0.022	51.0	54.9	0.344
5. History of falls (fell ≥ 2 times during the past 6 months) (%)	3.9	3.9	1.00	4.9	4.9	1.00
6. Housing style (first floor 1.5 m or higher from ground: Traditional Thai style (%)	81.4	80.4	1.00	86.3	86.3	1.00
Risk (≥ 4 score) (%)	52.9	32.4	<0.001	60.8	53.9	0.016

* *statistical significance set at 0.05, McNemar’s test*

**Table 4:** Comparison between intervention and control group of fall risk, 6 months intervention

***Fall-Risk Assessment Tool (Thai-FRAT)***	***Intervention (n=102)***	***Control (n=102)***	***P-value***
1. Females (%)	66.7	67.6	0.881
2. Visual impairment (unable to read more than half of the letters in the 6/12 line of the Snellen chart) (%)	44.1	42.2	0.777
3. Balanced impairment (unable to take full tandem for 10 seconds) (%)	32.4	46.1	0.045 ^*^
4. Medicine usage (taking 4 medications of any kind) (%)	39. 2	54.9	0.025 ^*^
5. History of falls (fell ≥ 2 times in past 6 months) (%)	3.9	4.9	0.733
6. Housing style (first floor 1.5 m or higher off ground: Traditional Thai style (%)	80.4	86. 3	0.260
Risk (≥ 4 score) (%)	32.4	53.9	0.002 ^*^

* statistical significance set at 0.05, Chi squared test

## Discussion

The subjects were recruited from individuals who underwent meeting and health promotion in their community and were divided into 2 groups. No significant differences were found between the 2 groups in any of the baseline values of the socio-demographic characteristics or health status. Therefore, the comparison of the 2 groups was assumed to be valid as a study design.

### Prevalence of fall risk among the elderly at based line

More than half of the all sampled elderly were at risk of a fall. The majority of the respondents at risk of a fall were female. The rate of falls related injuries among older women higher than men which affected to their health and also found the rate of fracture in older women was 2.2 times to older men (18). Therefore, information about sex differences vis-à-vis locations, circumstances, and events, preceding fall-related injuries, are needed to identify high-risk situations, behaviors, and develop targeted fall prevention strategies.

Forty percent of respondents in the current study (in both the intervention and the control groups) had some visual impairment. In Thailand, 65.8% of the elderly had moderate visual impairment (20/70 to 20/200) and the common eye diseases among Thai elders were cataracts and glaucoma (19). Visual impairment increased risk of falls and fracture in old age (20). Similar to the study in China, visual impairment was significantly related to falls in old age (AOR, 1.98; 95% CI, 1.02–4.32; *P*<0.001) (21). In addition, aging process and multifocal glasses were the risks for falls in old age. The contrast sensitivity and depth perception were decreased dramatically by the aged while multifocal glasses would increase the risk of falls due to the near vision lenses impair distance contrast sensitivity and depth perception in the lower visual field which reduces the ability of the old age to detect environmental hazards (22).

Half of all sample had balance impairment, which was less than the previous study in Chumporn Province, Thailand (63.6%) (23). However, gait and balance were important factors related to fall among the old age. There were multiple factors related gait and balance, and notably for arthritis and orthostatic hypotension elders (24).

The majority (63.6%) of the elderly in the current study had taken 4 medications on a daily basis within the past 6 months, according to a doctor’s diagnosis or self-prescription; which was less than the 63.6% reported (23). The more drugs that the elderly take, the greater their risk of falls (25). Poly-pharmacotherapy (≥ 4 drugs) is a known risk factor for falls when associated with a daily regimen of a risk medication (OR 2.157 CI 95% 1.447–3.217) (26). Normally, the elderly were easy to access psychotropic drugs in a community or residential care setting such as benzodiazepines (particularly, long-acting agents), antidepressants, and antipsychotic drugs. The use of benzodiazepines is the strongest predictor for falls among the elderly (27).

History of falls in the current study was 3%–4% who fell 2 or more times in the past 6 months, which is lower than that found 24.4% had fallen 1 or more times during the past 6 months (23). This prevalence is comparable to the study in China where the prevalence of recurrence is 4.75% (28). Two or more falls is the main determinant for recurrent falls (29).

Due to Traditional Thai housing style popularly has a two storey single-family home which stairways are the risk of fall (30,31). The climbing up stairs difficulty was associated with poor balance and grip strength while climbing down stairs difficulty had opportunity for more fall. In addition, climbing up and down stairs were associated with leg claudication, fear of falling, nonneurologic gait abnormalities, and slow gait (31).

Half the respondents were at risk of a fall. The prevalence of risk in the intervention group was 52.9% compared to 60.8% in the control group. This constitutes a relatively high prevalence compared to the 44.4% reported in a study in Chumporn Province, Thailand (23). By comparison, in a Latino population using a different tool, 52% were found to have 5 or more of the 10 surveyed risk factors for falling (32). These findings may contribute to earlier identification of high-risk fallers and interventions for fall prevention.

### Effect of Health Education program for fall risk prevention

This study followed 6 steps for fall prevention: (i) determining the preparedness of the system to serve the population at risk of falls, (ii) staff training and education, (iii) fall-risk assessment, (iv) intervention and management of clinical outcomes, (v) reimbursement of fall prevention services and (vi) assessment of clinical effectiveness (33). Health education focuses on the buddy technique with using the two-way communication and focus on peer support which aims to encourage home-based exercise, raise awareness of use and interactions of medicines, make modifications to the home environment and promote new behavioral factors for fall risk prevention among the elderly. We found the proportion of fall-risk was slightly decreased in the intervention group but not proportionally different in the control.

The home base exercise program aims to increase muscle strength, improve balance and also keeps joints, tendons, and ligaments flexible which stimulated daily exercise by the elder’s buddy at a convenient place for 15–20 min a day. The health education by buddy/peer system was effective to a positive attitude vis-à-vis the continuance of exercise at home 20 min/session at least 3 times a week (34). The sufficient exercise was significantly effective in enhancing muscle strength, ankle flexibility and balance (35).

Due to the group dialogue and the buddy intervention, the elderly had the opportunity to share their experiences of falls, fall risk and use of medications. In these activities, peer support provided social companionship, instrumental aid, as well as emotional comfort, helping to release pressure, reduce depressive feelings, raise awareness the of medicines and buffer the ill effects of stressors (36). Moreover, home base exercise program intervention might help the elderly reducing depression (35). The results of this study may result in less use of sedatives and psychotropic medications.

In terms of overall fall-risk, proportion of fall-risk decreased significantly in the intervention group compared to the control group. In a similar study of the elderly in Taiwan, intervention education regarding fall prevention significantly improved prevention knowledge, Health Beliefs and cues to action, fall prevention behavior and the times of falls at home and in the community (37).

The possible barriers to learning among the elderly due to aging (i.e. sensory loss, mental illness and chronic diseases) could affect the intervention outcomes (13). Since ours was a community intervention study, it was not possible to allocate subjects randomly into the control and intervention groups.

## Conclusion

A health education program on fall risk prevention improved balance impairment and medicine use among the elderly. Similar areas should be included in this health education fall risk prevention program. Rural areas are recommended for further study of a fall risk prevention program.

## Ethical considerations

Ethical issues (Including plagiarism, informed consent, misconduct, data fabrication and/or falsification, double publication and/or submission, redundancy, etc.) have been completely observed by the authors.
